# The prolongation of somatic support in a pregnant woman with brain-death: a case report

**DOI:** 10.1186/1742-4755-3-3

**Published:** 2006-04-27

**Authors:** João P Souza, Antonio Oliveira-Neto, Fernanda Garanhani Surita, José G Cecatti, Eliana Amaral, João L Pinto e Silva

**Affiliations:** 1 Intensive Care Unit Department of Obstetrics and Gynecology, School of Medical Sciences, State University of Campinas, Campinas, São Paulo, Brazil; 2 Obstetrics Unit, Department of Obstetrics and Gynecology, School of Medical Sciences, State University of Campinas, Campinas, São Paulo, Brazil

## Abstract

**Background:**

Medical literature has increasingly reported cases of maternal brain death during pregnancy. This is a rare situation which demands the decision and, depending on the gestational age, the implementation of a set of measures to prolong the homeostasis of the human body after brain death for the purpose of maintaining the foetus alive until its viability.

**Case presentation:**

A 40 year old woman suffered an intracranial haemorrhage during the 25^th ^week of pregnancy. Despite neurosurgical drainage of a gross intraparenchymatous haematoma, the patient developed brain death. Upon confirmation of this diagnosis, she received full ventilatory and nutritional support, vasoactive drugs, maintenance of normothermia, hormone replacement and other supportive measures required to prolong gestation and improve the survival prognosis of her foetus. All decisions regarding the patient's treatment were taken in consensus with her family. She also received corticosteroids to accelerate foetal lung maturity. During the twenty-five days of somatic support, the woman's condition remained stable; however, during the last seven days the foetus developed oligohydramnios and brain-sparring, which led the medical team to take the decision to perform a Caesarean section at that moment. After delivery, the patient's organs were removed for donation. The male infant was born weighing 815 g, with an Apgar score of 9 and 10 at the first and fifth minutes, respectively. The infant was admitted to the neonatal intensive care unit, but did not require mechanical ventilation and had no major complications. He was discharged at 40 days of life, with no sequelae and weighing 1850 g.

**Conclusion:**

These results are in accordance with findings from previous studies and case reports suggesting the appropriateness and safety of extended somatic support during pregnancy under certain circumstances. They also suggest the need for prompt diagnosis of brain death before the occurrence of physiological degeneration, rapid evaluation of foetal status and the decision of the family together with the medical team to prolong maternal somatic support. The occurrence of maternal brain death is a tragedy, but it may also represent a challenging opportunity to save the life of the foetus and, in addition, permit donation of the maternal organs.

## Background

The medical literature has reported with increasing frequency the occurrence of maternal brain death during pregnancy [1]. Nevertheless, this event remains a rare situation for most obstetricians and critical care specialists. There is a paucity of data regarding the frequency of this event, but one organ procurement organization reported an incidence of seven pregnant women among 252 deceased female organ donors (2.8%) identified after brain death [2]. Although rare, when maternal brain death occurs during pregnancy, a dilemma is faced: should the foetus be delivered immediately, should it be allowed to die when its mother's artificial life-support system is disconnected, or should a conservative approach focused on the maintenance of maternal homeostasis be attempted in order to achieve greater foetal maturity? [1]

These questions have been answered empirically, based on the experience of a few cases of maternal brain death during pregnancy reported in the medical literature during the last quarter of a century. Nevertheless, the number of unsuccessful cases that were not reported is unknown [1,3]. Among the eleven reported cases, the longest period of support was 15 weeks and 2 days [4] and there have been only two cases of successful delivery associated with maternal organ donation [1]. According to the rationale of these reports, when gestational age is compatible with optimal neonatal survival, usually above 32–34 weeks, immediate delivery is appropriate. If the pregnancy is less than 20 weeks, it would be acceptable to discontinue any artificial measures to prolong maternal life, although one case has been reported in the literature in which successful somatic support began at 15 weeks of gestational age [4]. In this context, the strongest indication for somatic support during the pregnancy is when maternal brain death occurs between the 20^th ^and the 32^nd ^week of gestational age.

In this report, a case of maternal brain death at 25 weeks of gestation is described. Somatic support was provided for 25 days and the complexity of this procedure is discussed.

## Case presentation

A 40 year old woman was admitted to the obstetrics ward during the 25^th ^week of gestation to adjust her anticoagulation regimen. She was gravida 2, with one previous miscarriage and had a metallic mitral valve prosthesis as a result of a childhood episode of rheumatic fever. Except for her cardiac disease, which was under control, she led a normal life and was employed as a social worker. This woman discovered that she was pregnant in the first trimester of gestation, and without consulting her physician, stopped using her anticoagulants. She was late in initiating antenatal care and consequently her referral to a specialized antenatal clinic was also delayed. When she was in the 25^th ^week of gestation, she reached the third level of care and was admitted to the obstetrics ward where she was prescribed 10,000 units of unfractionated heparin twice a day. During her third night in the hospital, after receiving subcutaneous heparin for almost 72 hours (at that time the dose had been increased to 12,000 units twice a day), she developed headache, nausea, vomiting and mental confusion. At that time, physical examination revealed a stiff neck, but no focal neurological deficits were noted. The patient's pupils showed isocoria and were reactive to light, and she had a Glasgow coma score of 11. During this initial phase of hospitalization, her blood pressure and pulse remained within the normal range. APTT (Activated Partial Thromboplastin Time) was 57 seconds. A computerized tomography scan displayed an intraparenchymatous haematoma with bloody obliteration of the ventricles. The patient underwent neurosurgical drainage of this lesion and was admitted to the obstetrical intensive care unit, where postoperative care and ventilatory support was continued. During the following hours, her neurological status deteriorated progressively and on the following day, the patient appeared to have gone into a non-reactive coma with no sign of brainstem activity. Maternal brain death was confirmed by a transcranial Doppler scan of the cerebral arteries. At that time, foetal weight was estimated at 660 g according to ultrasound evaluation. After the diagnosis of brain death, the situation was explained to the woman's family, who informed the medical team that the deceased woman had legally registered her desire to posthumously donate her organs. In view of the deceased woman's wishes and the status of the foetus, a conservative approach was adopted by the medical team, and the necessary measures to prolong maternal homeostasis were implemented.

We assumed that brain death would have resulted in a condition of panhypopituitarism caused by loss of the hypothalamic-pituitary axis function. Accordingly, enteral replacement of thyroid hormone and cortisol (prednisone) was carried out. The patient also developed a persistent state of hyperglycemia that was treated with continuous intravenous insulin. *Diabetes Insipidus *was treated with enteric desmopressin (DDAVP), and diuresis and serum sodium were used to titrate the dose of DDAVP.

The patient developed mild hypotension, which was initially treated with crystalloid and colloid replacement to maintain central venous pressure between 14 and 18 centimetres of water, plus low doses of norepinephrine. A moderate but persistent bradycardia was initially treated with dopamine and later with low doses of epinephrine. Pressure-limited mechanical ventilation was used to manage mild respiratory alkalosis (CO2 = 32 mmHg) and an arterial oxygen saturation greater than 94%.

Basal energy expenditure (BEE) was calculated using the Harris-Benedict formula (BEE = 655 + [9.6 × weight(kg)] + [1.8 × height(cm)] - [4.7 × age(yr)]), multiplied by 1.25 (stress factor for pregnancy), adding 300 Kcal for a single foetus and deducting 15% from the BEE to compensate for the reduction in metabolism associated with the state of brain death [1]. Enteral nutrition was used to provide the total daily calorie requirement.

Hypothermia was a major feature during this period and was treated with passive heating using an air heater and blankets to maintain temperature > 36°C. After ten days of somatic support, the patient developed ventilation-associated pneumonia, which was treated with ceftriaxone, and a tracheotomy was performed to facilitate bronchial hygiene and mechanical ventilation.

Foetal well-being was thoroughly evaluated by electronic foetal heart rate monitoring, foetal Doppler velocimetry and biophysical profile. Betamethasone was used to accelerate foetal lung maturity. During a total of twenty-five days of prolongation, the maternal organism remained mostly stable. Nevertheless, in the last week of prolongation progressive oligohydramnios was detected in the foetus and a Doppler test revealed centralization (brain sparring). Foetal status helped in reaching the decision to perform a Caesarean section at that moment. It is possible that, even at the low doses used, the vasoconstrictors may have contributed to the functional placental insufficiency by constricting the uterine vessels, resulting in placental hypoperfusion. A male infant was born weighting 815 g, with an Apgar score of 9 and 10 in the first and fifth minutes, respectively. Following delivery, the premature newborn was admitted to the neonatal intensive care unit. He required no mechanical ventilation and had no major complications. Some hours after delivery, the maternal organs were removed for donation. The baby was discharged from hospital weighing 1850 g at 40 days of life. Re-evaluation of the infant during a follow-up visit at 90 days of life revealed normal development and no detectable sequelae.

## Discussion

According to an electronic search (MedLine and SciELO) of the medical literature, this is the twelfth reported case of maternal brain death during pregnancy, and the third involving organ donation. Despite this paucity of data, in the United States alone there were 2,642 deceased female organ donors in 2005 [4]. Estimating that 2% of these women were pregnant when their organs were donated, a figure that is lower than the number actually reported [2], there would be 53 pregnant women succumbing to brain death among US organ donors annually. If we consider that half the patients with brain death were organ donors and that a tenth of the cases of brain death in the world are concentrated in the US, there would be approximately 1,060 pregnant women with brain death annually in the world. Although these calculations of the numbers of cases of brain death during pregnancy are mere estimates, a guide for the management of these very complex cases seems justifiable.

From an ethical standpoint, some concerns have been raised regarding this situation [1,5,6]. The decision with respect to the artificial prolongation of the deceased mother's vital function is made by the mother's next-of-kin, except if the foetus's biological father was not married to the deceased mother. In this case, since the decision affects the interests and well-being of the foetus, the biological father may play a major role. Certainly, one of the determinant factors in this decision-making process is a prior statement by the mother with respect to organ donation. If the deceased mother is an organ donor, then prolongation of her vital function is more easily justified from an ethical point of view, since the foetus would be the first to benefit from receiving the donation of the mother's organic function. This decision may also be supported by an ethics committee, with the full agreement of the family and the documented opinion of the woman, if available.

From the financial point of view, extended somatic support is expensive; however, the savings in neonatal intensive care unit costs must also be taken into consideration. In the case reported here, the twenty-five days of somatic support allowed corticosteroids to be given and resulted in an increase in foetal weight from 660 g to 815 g. Although the foetus was still very premature at delivery, we believe that these additional three weeks of intrauterine time contributed positively to foetal outcome and to a reduction in the duration of neonatal hospitalization and costs.

From the technical point of view, the set of measures is complex, requiring full intensive care support and additional efforts to compensate for the absence of a functioning brainstem. In this way, we believe that the essentials components of a successful and extended somatic support are the rapid onset of intensive care, hormone replacement and thermal maintenance.

## Conclusion

This report is in agreement with previous studies and case reports, suggesting the appropriateness and the safety of extended somatic support during pregnancy under certain circumstances. It also emphasizes the need for prompt diagnosis of brain death before the occurrence of physiological degeneration, rapid evaluation of foetal status with respect to its viability and well-being, and the decision of the family together with the medical team to prolong maternal somatic support. Although the occurrence of maternal brain death is a tragedy, it may also represent an opportunity to save the life of the foetus and in addition permit donation of the maternal organs.

## Competing interests

The author(s) declare that they have no competing interests.

## Authors' contributions

JPS and AON were responsible for the medical care at the intensive care unit. FGS, EA, JGC and JLPS were responsible for the obstetrical care of the woman and for taking decisions regarding the appropriate procedures. JPS performed the literature review and wrote the first draft of the report. JGC reviewed the first draft of the paper and made appropriate changes. All authors provided suggestions for the manuscript, read it carefully, agreed on its content and approved the final version.
